# iPSC modeling of severe aplastic anemia reveals impaired differentiation and telomere shortening in blood progenitors

**DOI:** 10.1038/s41419-017-0141-1

**Published:** 2018-01-26

**Authors:** Dario Melguizo-Sanchis, Yaobo Xu, Dheraj Taheem, Min Yu, Katarzyna Tilgner, Tomas Barta, Katja Gassner, George Anyfantis, Tengfei Wan, Ramu Elango, Sameer Alharthi, Ashraf A. El-Harouni, Stefan Przyborski, Soheir Adam, Gabriele Saretzki, Sujith Samarasinghe, Lyle Armstrong, Majlinda Lako

**Affiliations:** 1 Institute of Genetic Medicine, Newcastle University, Newcastle, UK; 2 The Ageing Biology Centre. Institute for Cell and Molecular Biosciences, Newcastle University, Newcastle, UK; 3 Princess Al Jawhara Al-Brahim Center of Excellence in Research of Hereditary Disorders, King Abdulaziz University, Jeddah, Saudi Arabia; 4 Department of Bioscience, Durham University, Durham, UK; 5 Hematology Department, King Abdulaziz University, Jeddah, Saudi Arabia; 6 Department of Medicine, Duke University Medical Center, Durham, USA; 7 Department of Hematology, Great Ormond Street Hospital for Children NHS Foundation Trust, London, UK

## Abstract

Aplastic Anemia (AA) is a bone marrow failure (BMF) disorder, resulting in bone marrow hypocellularity and peripheral pancytopenia. Severe aplastic anemia (SAA) is a subset of AA defined by a more severe phenotype. Although the immunological nature of SAA pathogenesis is widely accepted, there is an increasing recognition of the role of dysfunctional hematopoietic stem cells in the disease phenotype. While pediatric SAA can be attributable to genetic causes, evidence is evolving on previously unrecognized genetic etiologies in a proportion of adults with SAA. Thus, there is an urgent need to better understand the pathophysiology of SAA, which will help to inform the course of disease progression and treatment options. We have derived induced pluripotent stem cell (iPSC) from three unaffected controls and three SAA patients and have shown that this in vitro model mimics two key features of the disease: (1) the failure to maintain telomere length during the reprogramming process and hematopoietic differentiation resulting in SAA-iPSC and iPSC-derived-hematopoietic progenitors with shorter telomeres than controls; (2) the impaired ability of SAA-iPSC-derived hematopoietic progenitors to give rise to erythroid and myeloid cells. While apoptosis and DNA damage response to replicative stress is similar between the control and SAA-iPSC-derived-hematopoietic progenitors, the latter show impaired proliferation which was not restored by eltrombopag, a drug which has been shown to restore hematopoiesis in SAA patients. Together, our data highlight the utility of patient specific iPSC in providing a disease model for SAA and predicting patient responses to various treatment modalities.

## Introduction

Aplastic Anemia (AA) is a rare and serious bone marrow disorder associated with hypocellular bone marrow and peripheral pancytopenia. Severe AA (SAA) is a subtype of the disease characterized by very low bone marrow cellularity of less than 25%, with significant morbidity and mortality^1^. AA occurs with peak incidences at the two extremes of life, in patients between the age of 10 and 25, and patients aged >60 years. Children with AA are more often treated with hematopoietic stem cell transplantation (HSCT) while adults are treated with either immunosuppressive therapy using anti-thymocyte globulin (ATG) and Cyclosporine or HSCT, if a matched donor is available^2^. Currently, 70–80% of cases are classified as idiopathic because their etiology is unknown. The remainder (15–20%) consists of constitutional bone marrow failure syndromes with the most common being Fanconi anemia (FA) followed by the telomeropathies such as dyskeratosis congenital (DC).

There are currently two proposed models of pathogenesis in idiopathic AA that could explain the characteristic marrow hypocellularity observed in this disorder. In model 1, an underlying abnormality of the hematopoietic stem cells (HSCs) may result in a predisposition to stem cell damage, as well as qualitative or quantitative defects of HSC production. In model 2, a deregulated immune response targets a normal HSC compartment. Strong evidence for an immune component to the pathogenesis of AA comes from the success of the immunosuppressive therapies in treating AA and associated clinical features, including aberrations in immune cell number, phenotype and function^2^. Evidence for an underlying stem cell/progenitor defect is derived from the observations of reduced hematopoietic progenitor cell numbers both at presentation and following successful therapy with ATG^3,4^, enhanced apoptosis of HSCs, upregulation of genes involved in cell death in hematopoietic progenitors obtained from AA patients^5–7^ and mutations in genes such as *PRF1*^8^, *MPL*^9^, *NSB1*^10^, *SBSD*^11^, *TERT*, *TERC*, and *TIF2*^12^ as well as polymorphisms in the *IFN gamma*^13^ found in a small number of patients.

Acquisition of cells from the bone marrow of AA patients is complicated by the paucity of hematopoietic stem/progenitor cells in these patients and the lack of mouse models which recapitulate the entire spectrum of AA^14–16^. Here we describe the generation of an iPSC-derived disease model for SAA consisting of iPSC lines derived from 1 pediatric and 2 young adult cases and 3 unaffected controls. These iPSC lines were differentiated to hematopoietic lineages and although no significant differences were observed in the ability to give rise to hematopoietic progenitors between control and SAA-iPSC, the later, showed a reduced potential to generate erythroid and myeloid cells, impaired proliferation and shorter telomeres when compared to unaffected controls.

## Results

### Generation of human iPSC from control and SAA patient fibroblasts

**Fig. 1:**
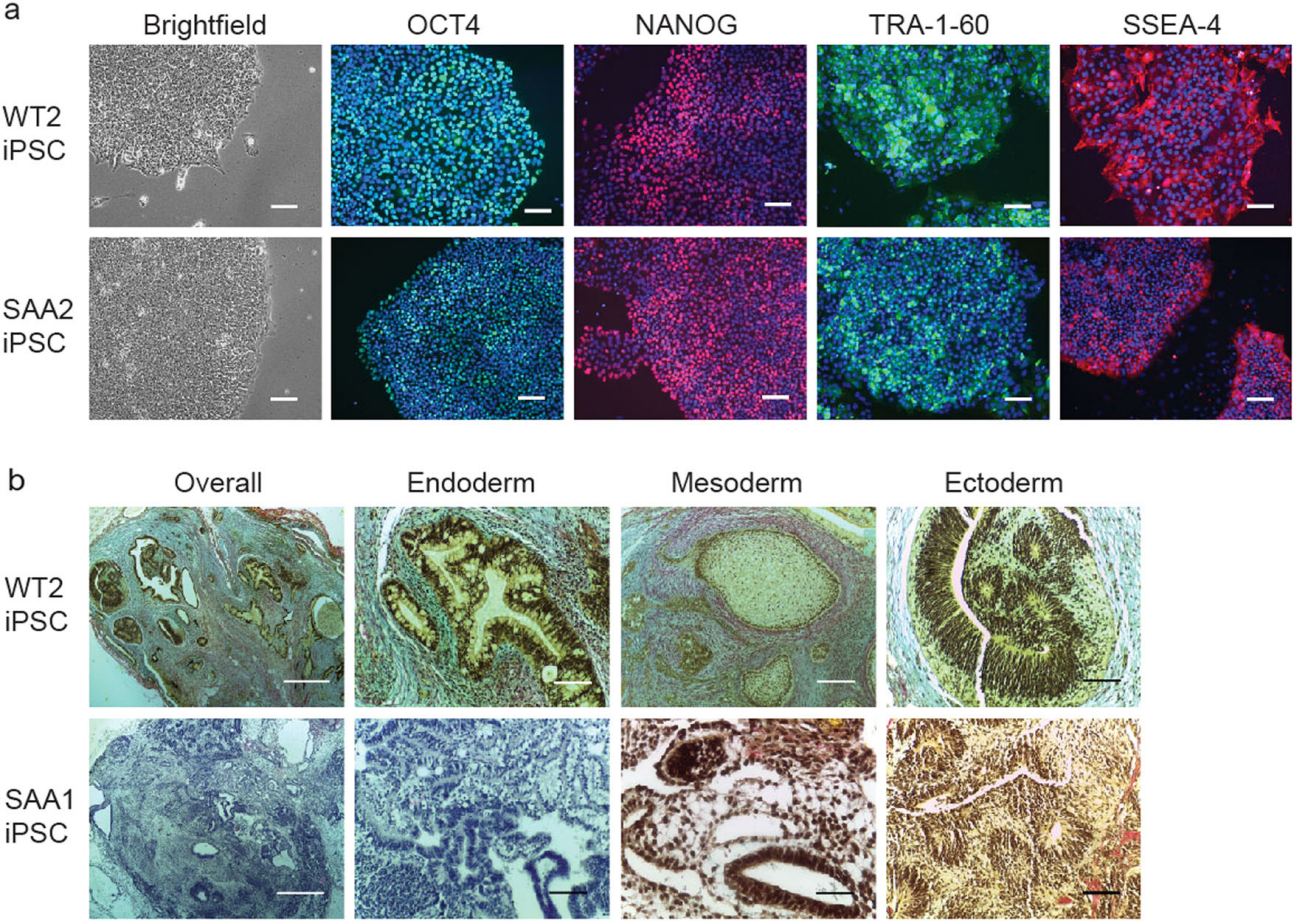
SAA-iPSC lines display in vitro hallmarks of pluripotency **a** Brightfield images of control and SAA-iPSC colonies displaying typical ESC-like morphology and staining of control and SAA-iPSC colonies with pluripotency markers. DAPI staining is shown in blue. Scale bars, 100 µm; **b** Histological analysis of representative teratomae generated for control and SAA-iPSC lines displaying trilineage differentiation. Scale bars, overall 500 µm, ectoderm 100 µm, mesoderm 200 µm, ectoderm 100 µm

To generate an in vitro disease model for SAA, dermal fibroblasts cells from 1 pediatric and 2 young adult cases of idiopathic SAA, showing severe and very severe clinical manifestation and no clinical stigmata of constitutional AA as well as three unaffected controls (indicated as WT throughout the manuscript) were transduced with the non-integrating RNA-derived Sendai virus (SeV) vector including the four Yamanaka reprogramming factors (*OCT4, SOX2, KLF4, c-MYC*) as previously reported^17^ (Table 1). SAA fibroblasts showed slightly reduced reprogramming efficiencies compared to unaffected controls (Table 1). No residual presence of the SeV transgenes was detected in any of the lines generated indicating that SAA-iPSC and control lines successfully maintained stable regulation of the endogenous pluripotent gene expression activated during reprogramming after the loss of SeV transgene expression (Supplementary Fig. 1). Cytogenetic analysis carried out by single nucleotide polymorphisms (SNP) array showed that the reprogramming process/prolonged culture of the iPSC lines did not induce any detectable genomic abnormalities with the exception of SAA1-iPSC in which two loss of heterozygosity events were identified (Supplementary Table 1). SAA-iPSC colonies displayed characteristic human ESC-like morphology and expression of pluripotent markers (Fig. 1a, Supplementary Fig. 2a and b). Similarly, in vivo assessment of pluripotency revealed that SAA-iPSC lines induced formation of teratomae containing cells belonging to all three germ layers (Fig. 1b, Supplementary Fig. 2c). These data indicate that SAA-iPSC and control-iPSC lines displayed characteristic features of fully-reprogrammed cells.

**Table 1:** Table providing detailed information regarding SAA patients used in this study and reprogramming efficiencies obtained for each control and patient

Patient ID	Age (years)	Gender	Phenotype	Reprogramming efficiency
WT1	Newborn	Male	Heathy	0.21%
WT2	51	Male	Healthy	0.29%
WT3	37	Female	Healthy	0.14%
SAA1	16	Male	Severe AA Responded to immunosuppressors	0.11%
SAA2	24	Male	Very Severe AA Responded to immunosuppressors Developed PNH and relapsed Failed with second course of horse ATG Successful matched unrelated HSCT	0.10%
SAA3	10	Female	Very Severe AA and autism Successful matched unrelated HSCT	0.11%

*AA* aplstic anemia, *PNH* paroxysmal nocturnal hemaoglobinuria, *ATG* anti-thymocyte globulin, *HSCT* hematopoietic stem cell transplantation

### Reduced colony-forming potential of SAA iPSC-derived hematopoietic progenitors

**Fig. 2:**
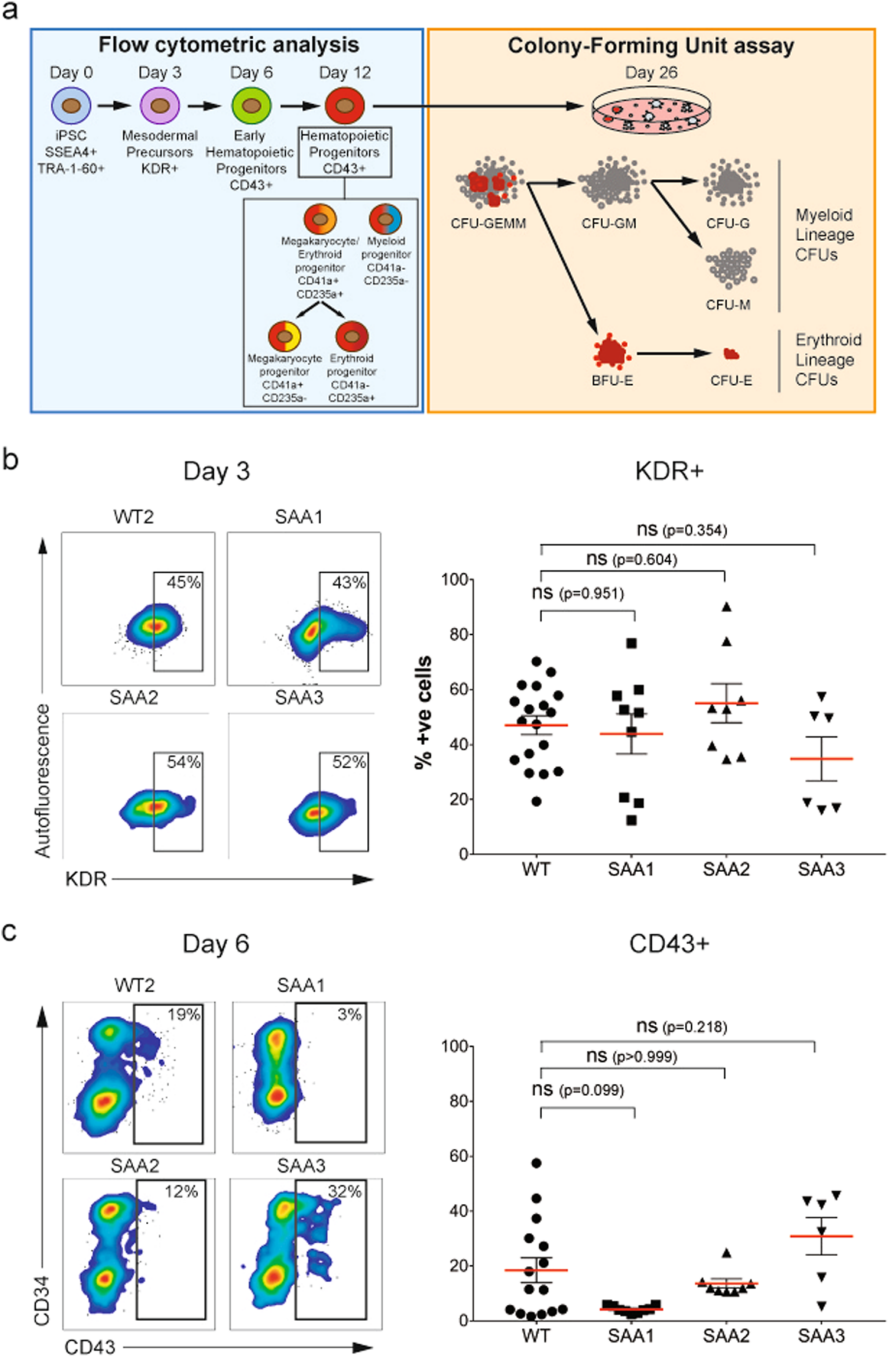
SAA-iPSC differentiation into mesodermal and hematopoietic progenitors **a** Schematic representation of the experimental design used to analyze the WT and SAA-iPSC hematopoietic differentiation capacity; **b** Representative images of flow cytometric analysis and scatter dot plot representation of KDR expression in WT and SAA cell lines on day 3. **c** Representative images of flow cytometric analysis and scatter dot plot representation of CD43 expression in differentiating WT and SAA cell lines on day 6. **b** One-way ANOVA with Dunnett’s multiple comparison test was used for statistical comparison between WT and SAA cell lines. **c** Kruskal-Wallis with Dunn’s multiple comparison test was used for statistical comparison between WT and SAA cell lines. **b**, **c** Data is presented as mean of at least 3 independent experiments ± S.E.M. Data for all control cell lines is averaged in one group (WT)

To investigate the hematopoietic differentiation potential of the SAA-iPSC lines, all patient specific and control iPSC were differentiated using a method previously described by Olivier et al.^18^. Early stages of mesoderm induction from iPSC cultures were monitored on day 3 of differentiation by expression of KDR (FLK1)^19^. Generation of the first hematopoietic progenitors was detected at day 6 using the CD43 pan-hematopoietic marker^20,21^. The emergence of hematopoietic progenitors (CD43+) and the subtypes of hematopoietic progenitors including megakaryocyte progenitors (CD41a+CD235a−), erythroid progenitors (CD41a-CD235a+), megakaryocyte/erythroid progenitors (CD41a+CD235a+) and myeloid progenitors (CD41a-CD235a−) was assessed by flow cytometric analysis throughout the differentiation time course^20^ (Fig. 2a). To identify the sources of variation that could affect the ability to generate hematopoietic progenitors, different variables such as differentiation experiment, passage number, clonal and donor cell origin (genetic background) were compared using the control-iPSC lines by flow cytometric analysis^22^ (Supplementary Fig. 3a). None of these parameters showed a statistically significant difference in the percentage of CD43 positive cells at day 12 (Supplementary Fig. 3b–d). Hence, one clone from each patient and control was used throughout this study. To enable comparison of data from each patient against all three controls, the latter were pooled together, averaged and shown as WT throughout the manuscript.

**Fig. 3:**
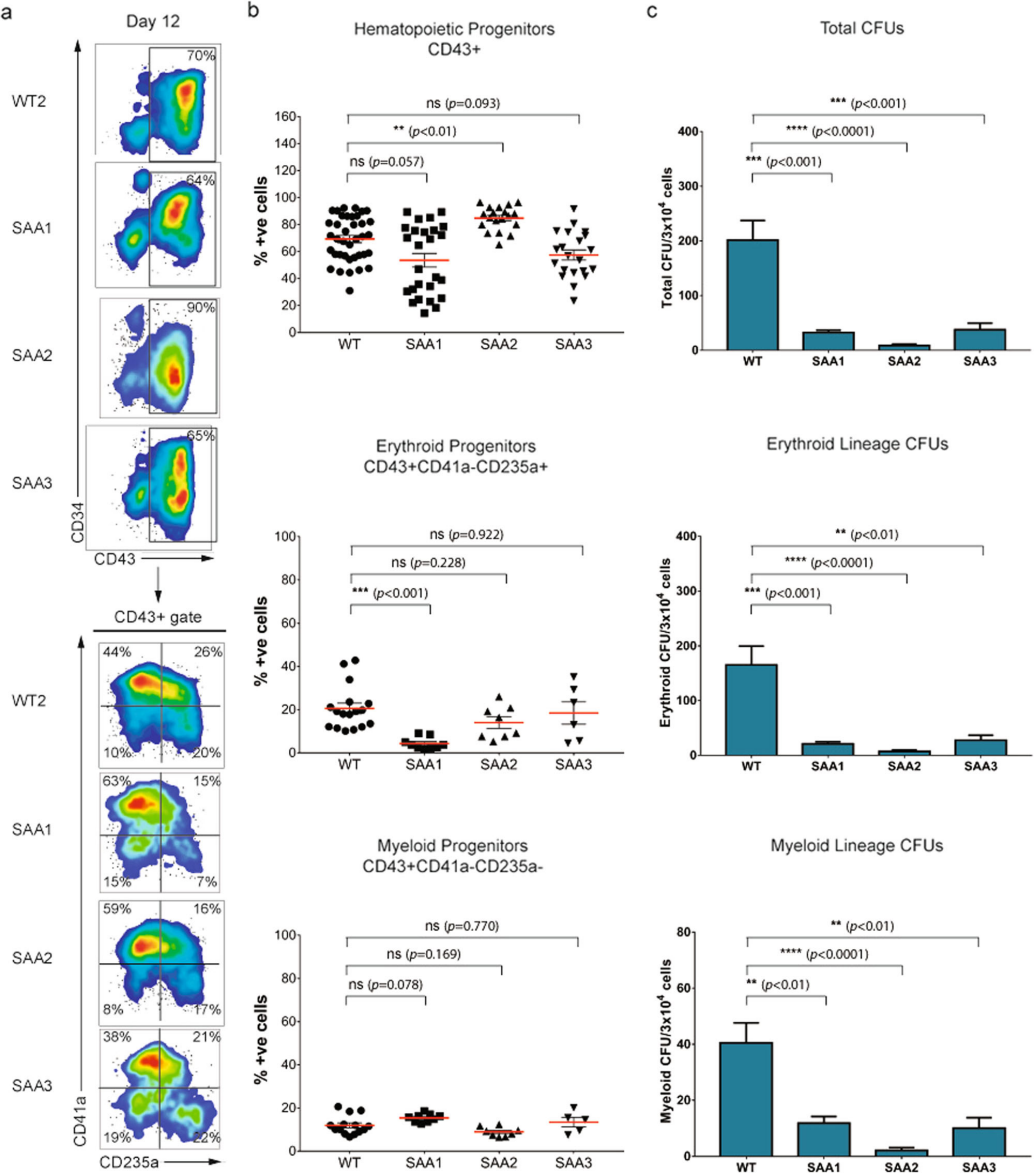
**SAA-iPSC-derived-hematopoietic progenitors show a reduced colony-forming potential** **a** Representative images of flow cytometric analysis of CD34 and CD43 expression and CD41 and CD235 expression on CD43+ population in WT and SAA cell lines on day 12 of differentiation; **b** Analysis of percentages of hematopoietic progenitors (CD43+), erythroid progenitors (CD43+CD41a−CD235a+) and myeloid progenitors (CD43+CD41a−CD235a-) in WT and SAA cell lines on day 12. Kruskal-Wallis with Dunn’s multiple comparison test was used for hematopoietic progenitors and one-way ANOVA with Dunnett’s multiple comparison test was used for erythroid and myeloid progenitors for statistical comparison between WT and SAA cell lines in; **c** Analysis of total colony-forming units (CFUs), erythroid-lineage CFUs and myeloid-lineage CFUs generated from WT and SAA-iPSC based hematopoietic progenitors on day 12. One-way ANOVA with Dunnett’s multiple comparison test was used for statistical comparison between WT and SAA cell lines; **b**, **c** data is presented as mean of at least three independent experiments ± S.E.M. Data for all control cell lines is averaged in one group (WT)

Analysis of mesodermal induction in SAA-iPSC lines at day 3 revealed that patient cell lines showed similar frequencies of KDR+ compared to control-iPSC lines (Fig. 2b). No significant reduction in the potential to generate CD43+ hematopoietic progenitors was observed in any of the SAA-iPSC cell lines compared to unaffected controls at days 6 and 12 (Figs. 2c and 3b); however one of the SAA patient iPSC (SAA1) showed a statistically significant reduction in the potential to generate erythroid progenitors (CD43+CD41a−CD235a+) (Fig. 3b).

To assess the colony-forming potential of the SAA-iPSC cell lines, we performed colony forming unit (CFU) assay at day 12 by culturing iPSC-derived-hematopoietic progenitors in methylcellulose-derived media enriched with recombinant cytokines which promote the differentiation into committed erythroid progenitors (CFU-E and BFU-E), and myeloid lineage progenitors (CFU-G, CFU-M, CFU-GM, and CFU-GEMM) (Fig. 2a). All three SAA-iPSC-derived hematopoietic progenitors showed a significant reduction in the total number of CFUs indicating an impaired hematopoietic colony-forming capacity, including both erythroid and myeloid colony potential (Fig. 3c). Despite reduced hematopoietic colony number in SAA, no differences in types and ratios of colonies formed were observed (data not shown). Similar data were obtained at day 16 of differentiation (Supplementary Fig. 4), suggesting that the impaired hematopoietic colony forming ability was not due to a delay in differentiation process.

### Telomerase-independent impaired telomere elongation in SAA-iPSC cell lines and enhanced telomere attrition during hematopoietic differentiation

**Fig. 4:**
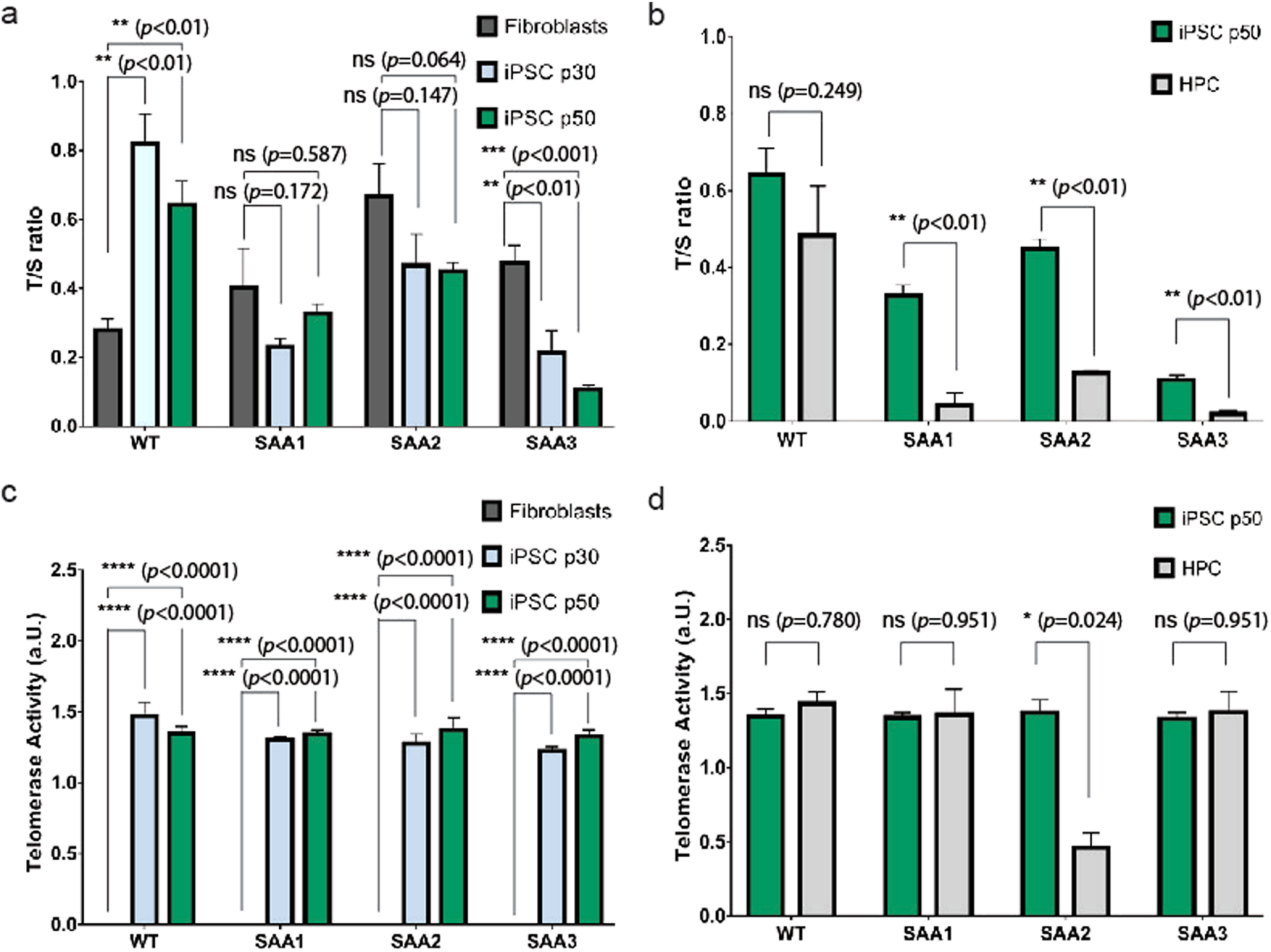
**SAA-iPSC exhibit deficient telomere elongation during reprogramming and telomere shortening upon hematopoietic differentiation** **a** Analysis of telomere length in parental fibroblasts (dark grey bars) and iPSC passage 30 (light blue bars) and 50 (green bars) in WT controls and SAA cell lines. One-way ANOVA with Tukey’s multiple comparison test was used for statistical comparison between fibroblasts and iPSC passage 30 and passage 50 (*p < 0.05); **b** Analysis of telomere length in iPSC passage 50 (green bars) and iPSC-based hematopoietic progenitors (HPC) differentiated from iPSC passage 50 (light grey bars) in WT controls and SAA cell lines. Multiple t-test using Holm-Sidak method was used for statistical comparison between iPSC passage 50 and HPC; **c** Analysis of telomerase activity in parental fibroblasts (dark grey bars) and iPSC at passage 30 (light blue bars) and 50 (green bars) in WT controls and SAA cell lines. One-way ANOVA with Tukey’s multiple comparison test was used for statistical comparison between fibroblasts and iPSC passage 30 and passage 50; **d** Analysis of telomerase activity in iPSC passage 50 (green bars) and iPSC-based hematopoietic progenitors (HPC) differentiated from iPSC passage 50 (light grey bars) in WT and SAA cell lines. Multiple *t*-test using Holm–Sidak method was used for statistical comparison between iPSC passage 50 and HPC. **a**–**d** data is presented as mean of at least 3 independent experiments ± S.E.M. Data for all control cell lines is averaged in one group (WT)

Excessive telomere attrition in highly proliferative cells such as hematopoietic stem and progenitor cells can lead to bone marrow failure^23^. Although the SAA patients used in this study did not display typical dyskeratosis congenita phenotype or mutations in *DKC1*, *TINF2*, *TERT* or *TERC* genes by exome sequencing analysis (Supplementary Tables 2 and 3), we decided to investigate the telomere dynamics in our SAA-iPSC model since it has been reported that one third of acquired AA patients present short telomeres in leukocytes^24–26^. Control-iPSC showed longer telomeres than parental fibroblasts due to telomere elongation during reprogramming, corroborating published reports^27^. No increases in telomere length were observed during the reprogramming of SAA fibroblasts (Fig. 4a). Furthermore, one of the patients (SAA3) showed continued telomere shortening during the reprogramming process (Fig. 4a), resulting in iPSC with telomeres which were significantly shorter than parent fibroblasts. The control iPSC lines did not show a significant telomere shortening during the 12 day differentiation time course to hematopoietic lineages; however all SAA-iPSC lines displayed a significant telomere attrition during the differentiation process, resulting in iPSC-derived-hematopoietic progenitors with significantly shorter telomeres than undifferentiated iPSC (Fig. 4b),corroborating data obtained with AA patient specific peripheral blood and bone marrow nucleated cells^26,28–30^.

We assessed telomerase activity in iPSC and iPSC-derived-hematopoietic progenitors by telomere repeat amplification analysis. Telomerase activity was significantly increased in iPSC when compared to parental fibroblasts indicating the expected up-regulation of telomerase activity during reprogramming of both control and SAA fibroblasts as reported from other studies^31^ (Fig. 4c). Likewise, analysis of telomerase activity in iPSC-derived-hematopoietic progenitors revealed no significant differences in telomerase activity compared to undifferentiated iPSC (Fig. 4d). Together, these data indicate impaired telomere elongation during the reprogramming of SAA fibroblasts, independent of telomerase activity measured by in vitro assays.

### Reduced proliferation capacity of SAA-iPSC-derived hematopoietic progenitors

**Fig. 5:**
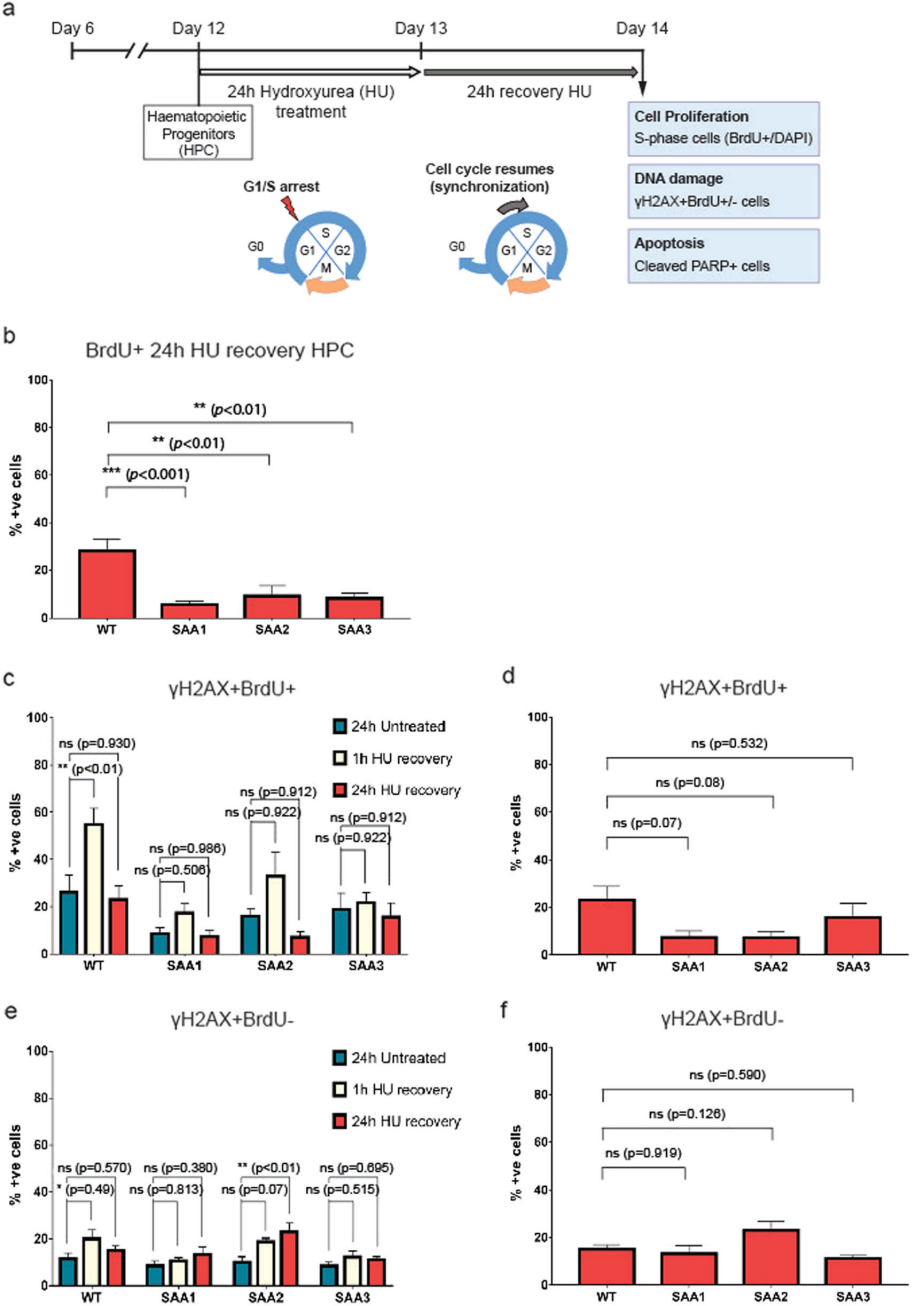
**SAA-iPSC-derived-hematopoietic progenitors show a reduced proliferation capacity** **a** Schematic of the experimental design used to analyze the proliferation, DNA repair capacity and apoptosis in SAA-iPSC-derived-hematopoietic progenitors; **b** Analysis of BrdU-incorporating cells in WT and SAA iPSC-derived-hematopoietic progenitors. One-way ANOVA with Dunnett’s multiple comparison test was used for statistical comparison between WT and SAA cell lines; **c** Analysis of γH2AX in BrdU+ cells in untreated (dark blue bars), 1 h after HU recovery (beige bars) and 24 h after HU recovery (red bars) iPSC-derived-hematopoietic progenitors. One-way ANOVA with Tukey’s multiple comparison test was used for statistical comparison between untreated cells and 1 h after HU recovery and 24 h after recovery; **d** Analysis of γH2AX in BrdU+ cells in WT and SAA iPSC-derived-hematopoietic progenitors. One-way ANOVA with Dunnett’s multiple comparison test was used for statistical comparison between WT and SAA cell lines; **e** Analysis of γH2AX in BrdU- cells in untreated (dark blue bars), 1 h after HU recovery (beige bars) and 24 h after HU recovery (red bars) iPSC-derived-hematopoietic progenitors. One-way ANOVA with Tukey’s multiple comparison test was used for statistical comparison between untreated cells and 1 h after HU recovery and 24 h after recovery,(*p < 0.05); **f** Analysis of γH2AX in BrdU- cells in WT and SAA iPSC-derived-hematopoietic progenitors. One-way ANOVA with Dunnett’s multiple comparison test was used for statistical comparison between WT and SAA cell lines. **b**–**f** data is presented as mean of at least 3 independent experiments ± S.E.M. Data for all control cell lines is averaged in one group (WT)

Progressive telomere shortening leads eventually to cell cycle arrest or cell death^32,33^. In view of this as well as the reduced capacity of SAA-iPSC to give rise to erythroid and myeloid cells, we investigated the proliferative capacity and apoptosis of the SAA iPSC-derived-hematopoietic progenitors. We synchronized the iPSC-derived-hematopoietic progenitors in G1/S phase by treatment with ribonucleotide reductase inhibitor hydroxyurea (HU) for 24 h. This was followed by culture in HU-free media and pulsing with 5-bromo-2-deoxyuridine (BrdU) for 1 h (Fig. 5a). Flow cytometric analysis of DNA content and incorporation of BrdU in control-iPSC-derived-hematopoietic progenitors indicated a high percentage of cells arrested in G1/S phase at 1 and 3 h post-release from HU due to depletion of deoxyribonucleotide pools (Supplementary Fig. 5a). These arrested cells reentered the cell cycle, progressed through S-phase and showed a similar cell cycle profile to that of untreated cells at 24 h post treatment (Supplementary Fig. 5a, b), indicating that iPSC-derived-hematopoietic progenitors require at least 24 h to restore a normal cell-cycle profile. We analyzed the proliferation rate of the synchronized control and SAA-iPSC-derived-hematopoietic progenitors (marked by CD43 expression) by comparing the percentage of BrdU-incorporating cells (S-phase) at 24 h post-release from HU. Interestingly, SAA-iPSC-derived-hematopoietic progenitors showed a significant reduction in the number of BrdU-incorporating cells (proliferative cells) compared with control counterparts, indicating a reduced proliferative capacity (Fig. 5b). To investigate whether apoptosis was increased in SAA-iPSC-derived-hematopoietic progenitors, we measured the presence of cleaved Poly (ADP-ribose) polymerase-1 (PARP) by flow cytometric analysis. Cleaved-PARP analysis of early apoptotic cells revealed a significant increase in the apoptosis of SAA-iPSC-derived-hematopoietic progenitors derived from one of the patients (SAA2) when compared to control cells (Supplementary Fig. 5c). However, no significant differences in apoptosis were observed between patients and controls after HU treatment (Supplementary Fig. 5d).

Induction of replicative stress by depletion of deoxyribonucleotide pools with long exposure to hydroxyurea leads to stalled replication forks and accumulation of DNA damage^34^. To address whether reduced hematopoietic potential of SAA-iPSC-derived-hematopoietic progenitors might be attributed to an impaired ability to repair DNA damage associated with replicative stress, we analyzed the percentage of DNA damage induced by HU treatment by flow cytometric analysis in proliferating and non-proliferating cells (Fig. 5c–f). To assess the reversibility of the DNA damage induced by HU exposure, we measured the formation of the phosphorylated histone variant H2AX (γH2AX) at 1, 3, 8, and 24 h after HU release in control-iPSC-derived-hematopoietic progenitors. This analysis indicated accumulation of γH2AX+ in BrdU+ cells at 1 and 3 h after HU release indicating accumulation of DNA damage in replication forks after HU replication block (Supplementary Fig. 5e). The γH2AX foci progressively disappeared showing similar levels of γH2AX levels to those of untreated cells at 24 h (Supplementary Fig. 5e). Thus, analysis of accumulation of DNA damage in proliferating (γH2AX+BrdU+) and non-proliferating (γH2AX+BrdU−) iPSC-derived-hematopoietic progenitors showed that control cell lines were able to repair the induced DNA damage after 24 h release from HU (Supplementary Fig. 5f, g).

To investigate the ability of SAA cell lines to repair DNA damage associated to replicative stress, we then analyzed the accumulation of γH2AX in proliferating (BrdU+) and non-proliferating (BrdU−) in SAA-iPSC-derived-hematopoietic progenitors at 1 and 24 h after HU release (Fig. 5c–f). No significant increase in the accumulation of γH2AX+BrdU+ was observed at 1 h post HU release in SAA-iPSC-derived hematopoietic progenitors (Fig. 5c), unlike the control counterparts which showed a significant accumulation of DNA damage at 1 h post HU release. Low levels of DNA damage observed in BrdU+ after replicative stress points to a reduced proliferation capacity likely due to a reduced formation of replication forks, in agreement with our results showing significantly reduced number of BrdU-incorporating cells (Fig. 5b). Thus, higher levels of γH2AX+BrdU+ cells were observed in control-iPSC-derived-hematopoietic progenitors compared to those in SAA cell lines, likely due to a higher number of replication forks, although the differences were not statistically significant (Fig. 5d). Interestingly, analysis of DNA damage in in non-proliferating (BrdU−) progenitors revealed a significant increase in the level of γH2AX at 24 h post HU release in one of patient-derived-hematopoietic progenitors (SAA2) compared to equivalent cells generated from the controls (Fig. 5e), suggesting an individual patient specific impaired ability to restore normal levels of DNA damage after HU treatment in non-proliferating iPSC-derived-hematopoietic progenitors. Notwithstanding this, the percentage of γH2AX+BrdU-cells in the hematopoietic progenitors derived from this patient iPSC line was not significantly higher when compared to equivalent cells generated from the unaffected controls (Fig. 5f). Together these data indicate that proliferating SAA-iPSC-derived-hematopoietic progenitors tend to accumulate less DNA damage soon after HU treatment most likely as result of their reduced proliferation. Furthermore, a subset of SAA-iPSC-derived-hematopoietic progenitors may be slower or have an impaired ability to restore DNA damage in the non-proliferative compartment. However, the overall level of DNA damage induced in response to replicative stress is not significantly different compared to control-derived-hematopoietic progenitors, excluding DNA damage accumulation as a key factor underlying the impaired hematopoietic differentiation of SAA-iPSC lines.

### Eltrombopag does not improve hematopoietic differentiation potential or enhance proliferation of SAA-iPSC-derived-hematopoietic progenitors

**Fig. 6:**
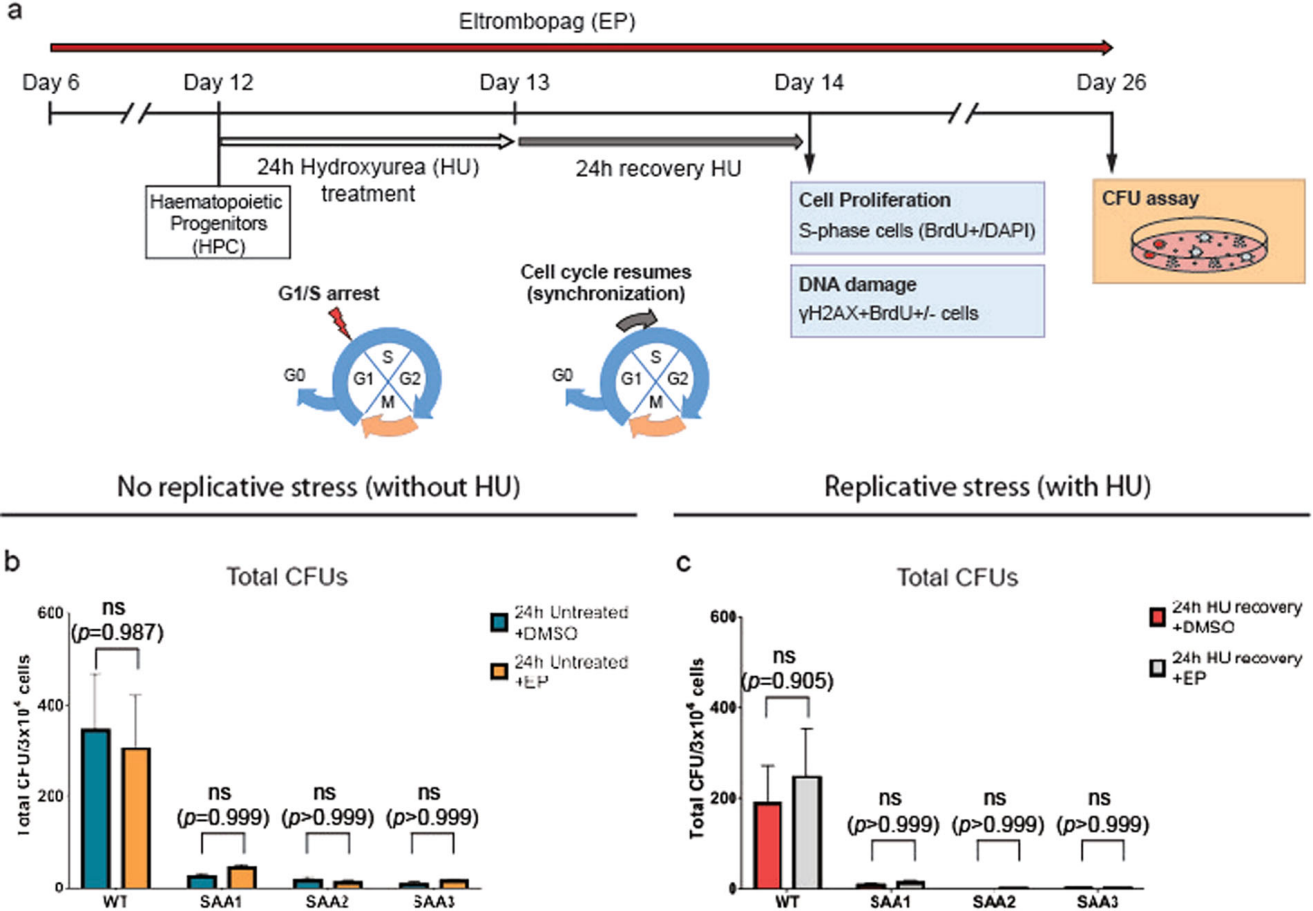
**Eltrombopag does not improve the colony-forming potential of SAA-iPSC-derived-hematopoietic progenitors** **a** Schematic of the experimental design used to analyze the effect of eltrombopag on the colony-forming potential, proliferation and DNA repair capacity in SAA-iPSC based hematopoietic progenitors; **b**–**d** Analysis of CFUs generated in DMSO (dark blue bars) and eltrombopag-treated (orange bars) iPSC-derived-hematopoietic progenitors in WT and SAA cell lines in non-replicative stress conditions; **b** Total CFUs, **c** erythroid-lineage CFUs, **d** myeloid-lineage CFUs. **e**–**g** Analysis of CFUs generated in DMSO (red bars) and eltrombopag-treated (grey bars) iPSC-derived-hematopoietic progenitors in WT and SAA cell lines under replicative stress conditions: **e** Total CFUs, **f** erythroid-lineage CFUs, **g** myeloid-lineage CFUs. **b**–**g** Multiple *t*-test using Holm-Sidak method was used for statistical comparison between DMSO and eltrombopag groups. Data is presented as mean of at least 3 independent experiments ± S.E.M. Data for all control cell lines is averaged in one group (WT)

Eltrombopag (EP) is a thrombopoietin (TPO) receptor agonist that promotes megakaryocyte maturation and platelet production without competing with endogenous TPO^35^. Beyond its role in megakaryopoiesis and platelet generation, TPO signaling has been also shown to be critical for HSC homeostasis and expansion in animal models and refractory SAA patients^36–38^. In view of these findings, we investigated whether the observed reduced colony-forming potential and proliferation of SAA-iPSC-derived-hematopoietic progenitors could be rescued by the addition of EP (10 µM) to the differentiation media (Fig. 6a). Addition of EP to TPO containing differentiation media induced a significant increase in the percentage of erythroid progenitors (CD43+CD235a+CD41a−) at the expense of megakaryocytic progenitors (CD43+CD235a-CD41+) compared to the control group containing only TPO in differentiation media (data not shown). These results are very similar to what has been reported in CD34+ bone marrow cells^39^ and indicate that EP is biologically active in our iPSC differentiation system.

To assess EP’s impact on SAA-iPSC differentiation, we added EP from day 6 of differentiation (Fig. 6a) in the presence and absence of HU and performed CFU assays at day 14 of differentiation. CFU assay results did not show significant differences between DMSO and EP-treated groups in the total number of colonies generated from SAA-iPSC-derived-hematopoietic progenitors cultured in absence of HU (Fig. 6b). Similarly, no differences were observed when EP was added in parallel to replicative stress inducing agent, HU (Fig. 6c).

**Fig. 7:**
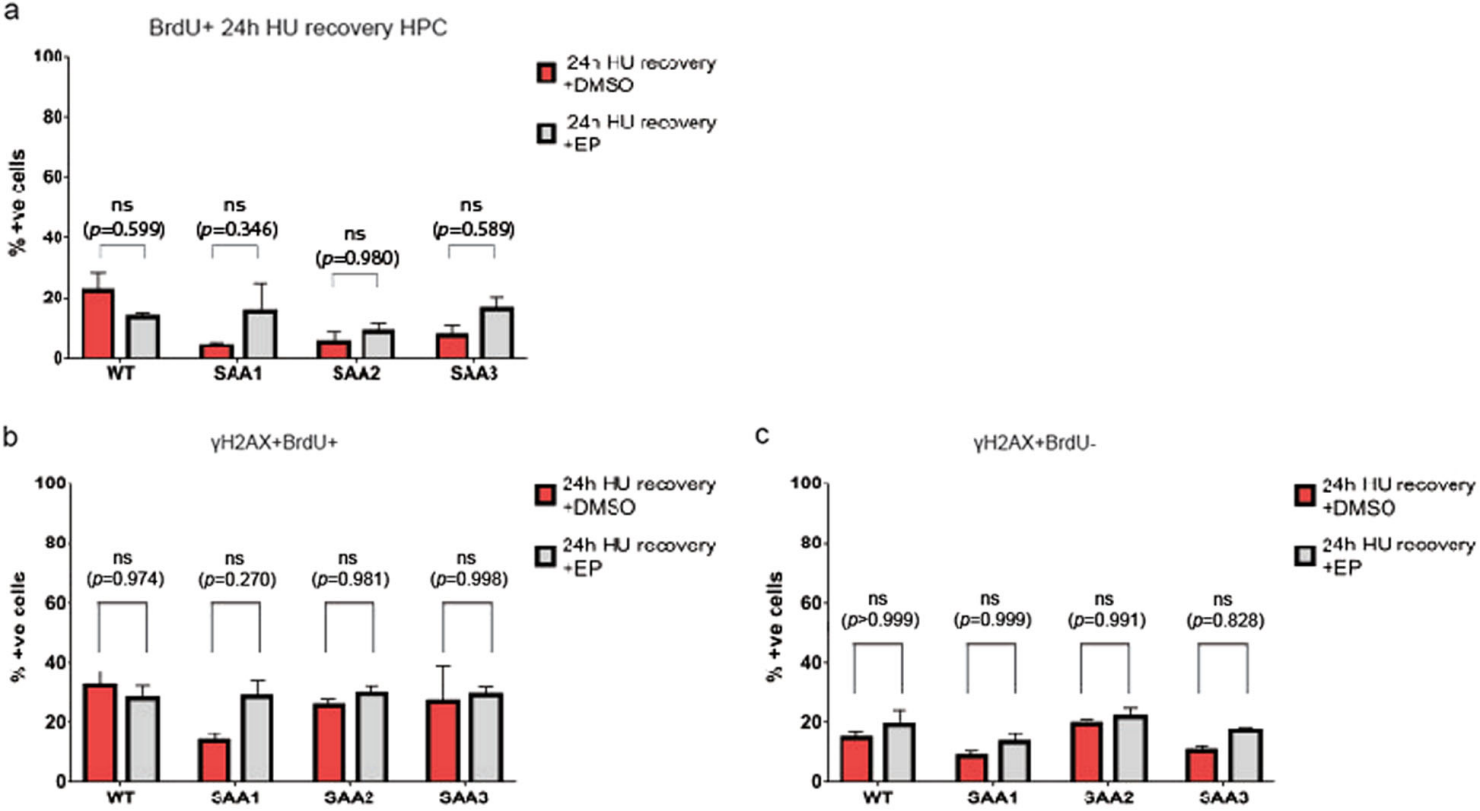
**Eltrombopag does not increase the proliferation capacity of SAA-iPSC-derived-hematopoietic progenitors** **a**. Analysis of BrdU-incorporating cells in DMSO (red bars) and eltrombopag-treated (grey bars) iPSC-derived-hematopoietic progenitors in WT and SAA cell lines; **b** Analysis of γH2AX in BrdU+ cells in DMSO (red bars) and eltrombopag-treated (grey bars) iPSC-derived-hematopoietic progenitors in WT and SAA cell lines; **c** Analysis of γH2AX in BrdU-cells in DMSO (red bars) and eltrombopag-treated (grey bars) in iPSC-derived-hematopoietic progenitors in WT and SAA cell lines. **a**–**c** Multiple t-test using Holm-Sidak method was used for statistical comparison between DMSO and eltrombopag groups. Data is presented as mean of at least 3 independent experiments ± S.E.M. Data for all control cell lines is averaged in one group (WT)

EP favors the proliferation and DNA double-strand break repair after *γ*-irradiation in human hematopoietic stem and progenitor cells^40,41^. To investigate whether EP is affecting the proliferative and DNA damage repair capacity of the SAA-iPSC-derived-hematopoietic progenitors under conditions of replicative stress induced by HU, flow cytometric analysis for BrdU incorporation and accumulation of γH2AX was carried out after EP treatment. No significant differences were observed in the percentage of BrdU+ cells, indicating that EP does not affect the proliferation of control or SAA-iPSC-derived hematopoietic progenitors (Fig. 7a). Similarly, no significant changes were observed in the percentage of proliferating and non-proliferating control-and SAA-iPSC-derived-hematopoietic progenitors with γH2AX foci (Fig. 7b, c). Together these data indicate that EP does not affect the proliferative capacity, DNA repair ability and colony forming potential of SAA-iPSC-derived-hematopoietic progenitor cells.

## Discussion

Studies of hematopoietic progenitor cell dysfunction in SAA have been difficult due to paucity of these cells in patient’s bone marrow samples. We have overcome this difficulty by using an iPSC disease model of three patients with SAA which has enabled us to study the phenotype of hematopoietic progenitors independent of the influence of the immune system.

SAA-iPSC lines were largely not impaired in their ability to generate hematopoietic progenitors; however the latter exhibited a much reduced erythroid and myeloid CFU ability accurately recapitulating the phenotype that defines SAA. The presence of reduced number and clonogenic capacity of bone marrow hematopoietic progenitors in AA patients has been reported by different studies^4,42,43^ and traditionally considered secondary to immune-mediated stem cell destruction^24^. Using our in vitro SAA iPSC model, we provide evidence for an impaired differentiation capacity in SAA hematopoietic progenitors in the absence of an immune system, which may be suggestive of a constitutional progenitor cell dysfunction. Genetic background has been reported as one of the major drivers of phenotypic variability in iPSC modeling^44–48^. We minimized this effect in the SAA-iPSC model by including multiple control cell lines in order to distinguish between phenotypic effects caused by disease-causing alterations and genetic background. Further experiments using a larger number of iPSC lines from relatives sharing similar genetic background are needed to fully understand the disease pathogenesis.

Functional reconstruction of telomeres and upregulation of telomerase activity during reprogramming represent a hallmark of induced pluripotency^27,31,49^. iPSC-derived modeling of telomeropathies associated with BMFs caused by mutations in telomerase-associated genes such as *TERC, TERT*, and *DKC* have shown defective telomere elongation in iPSC due to reduced telomerase function which impacts on the maintenance of pluripotent phenotype and hematopoietic differentiation capacity^50–52^. In SAA, only 10% of patients with short telomeres display known mutations in telomere pathway components suggesting that mutations in uncharacterized genes might have a role in the disease phenotype observed in these patients^24^. Our results revealed that SAA fibroblasts failed to elongate telomeres during the reprogramming process despite up-regulation of telomerase activity, pointing to an impaired telomere elongation during the reprogramming process that is not attributable to a defective telomerase activity. Similarly, iPSC-based hematopoietic progenitors in SAA cell lines showed excessive telomere shortening during hematopoietic differentiation. It is possible that the presence of short telomeres may be responsible for the impaired hematopoietic differentiation capacity we observed in our iPSC derived modeling of SAA^51^. Alternatively, the short telomeres can be a consequence of reiterative rounds of divisions carried out by a smaller number of proliferating hematopoietic progenitor cells in SAA patients as a compensatory mechanism to maintain homeostasis^15^. To be able to distinguish between these possibilities and investigate whether genetic predispositions play a role, whole genome analysis of a large number of SAA patients and their families need to be carried out. Our study included three patients only which precludes such large scale analysis, however exome sequencing analysis of patient’s dermal fibroblasts revealed the presence of deleterious mutations in telomere-associated genes such as *RPA2, NCL, POLD3, TEP1, YLMP1, PIF1*,* and ERCC4* (Supplementary Table 3). Interestingly, replication Protein A (RPA) has been reported to be necessary for telomere maintenance due to its role in unfolding of telomeric G-quadruplexes and preventing replication-fork stalling^53^ whereas *NCL* codifies for the RNA chaperone nucleolin that regulates the nuclear localization of telomerase^54^. Likewise, overexpression of *Ylpm1* in mouse embryonic stem cells leads to down-regulation of telomerase activity and telomere shortening resulting in reduced proliferation and hematopoietic differentiation ability^55^. Whilst these data point to existence of potentially deleterious mutations in telomere-associated genes in SAA patients, access to DNA samples from their related family members and larger cohorts of SAA patients is needed to confirm their involvement in SAA etiology.

EP is a non-peptide molecule mimetic to TPO that stimulates tri-lineage hematopoiesis in 40% of the SAA patients at 3–4 months^38,56^. However, the mechanism by which EP is promoting the generation of blood cells and impacting hematopoietic stem and progenitors in AA patients is not fully understood. We studied the ability of EP to rescue the impaired hematopoietic differentiation capacity in our SAA-iPSC model. SAA-iPSC-derived hematopoietic progenitors did not show a significant increase in the number of erythroid or myeloid-lineage CFUs, proliferative capacity or DNA damage repair capacity under conditions of replicative stress, upon adding EP during the differentiation process. Interestingly, it has been reported that EP failed to improve severe thrombocytopenia in patients with constitutional bone marrow failures syndromes including DC and Diamond-Blackfan-anemia^57^. Thus, this lack of response to EP by DC patients supports our hypothesis that constitutional defects in the telomere-associated genes may be at the root of the impaired hematopoietic differentiation observed in the SAA-iPSC-derived hematopoietic progenitors. As such it would be of interest to investigate if this group of patients will respond to other available therapies, for example danazol, although to date is unclear whether danazol related improvements are due to upregulation of telomerase activity through an increase in *TERT* expression or to elongation of telomeres^58,59^. Likewise, different authors have hypothesized that EP might be involved in immune cell function by modulating regulatory T cell function in SAA patients^38,60^ as observed in chronic idiopathic thrombocytopenic purpura patients treated with EP^61^. The lack of response to EP observed in our SAA-iPSC model suggests that EP may indeed have an underlying immune-regulatory function, which is separate to the impaired differentiation and telomere maintenance defect reported herein. In summary, our data provide strong evidence for usefulness of iPSC based disease modeling to replicate key phenotypes associated with SAA, facilitating the diagnosis of previously unidentified cases of constitutional SAA, and predicting patient specific response to various treatment modalities.

## Material and methods

### Generation of iPSC lines from SAA patients and healthy volunteers

Human fibroblasts from three healthy volunteers, one neonatal (Lonza, CC-2509) and two adult (Lonza, CC-2511), and three patients with pediatric SAA were cultured with Advanced Dulbecco’s Modified Eagle Medium (Thermo-Fisher, Waltham, MA, USA) containing 10% FBS (Thermo Fisher Scientific), 1% Glutamax (Thermo Fisher Scientific) and 1% penicillin/streptomycin (Thermo Fisher Scientific) at 37 °C and 5% CO_2_ in a humidified incubator. Fibroblasts were transduced using the Cytotune^TM^-iPS Reprogramming Kit (Thermo Fisher Scientific, A13780-01) according to manufacturer’s instructions. iPSC colonies were established on inactivated primary mouse embryonic fibroblasts feeder layer and then adapted to a feeder-free system, cultured on recombinant Vitronectin (Thermo Fisher Scientific) and in StemPro hESC SFM® media (Thermo Fisher Scientific) supplemented with 8ng/ml basic Fibroblast Growth Factor (Thermo Fisher Scientific), 1% penicillin/streptomycin and 0.1 mM 2-mercaptoethanol (Thermo Fisher Scientific).

### In vitro test of pluripotency

For immunocytochemistry analysis, iPSC colonies were fixed in 4% Formaldehyde (Sigma-Aldrich) and permeabilised with 0.25% Triton-X-100 (Sigma-Aldrich). Following treatment, cells were stained with mouse anti-human SSEA4-Alexa Fluor® 555 (Beckton Dickinson, BD; Franklin Lakes, NJ, USA, 560218) at 1:60 dilution, mouse anti-human TRA-1-60-FITC (Merck Millipore, Billerica, MA, USA, FCMAB115F) at 1:60 dilution, mouse anti-NANOG-AF647 (Cell Signaling Technologies, Danvers, MA, USA, 5448) at 1:150 dilution and goat antihuman OCT4 primary antibody (R&D, Minneapolis, MN, USA, AF1759) at 1:60 dilution. Secondary staining was performed using anti-goat IgG FITC (Sigma-Aldrich, F7367) at 1:200 dilution. Following treatment with the nuclear stain—DAPI (Partec), stained iPSC colonies were photographed using a Bioscience Axiovert microscope (Axiocam, CarlZeiss) in combination with the associated CarlZeiss software- AxioVision.

To assess the percentage of cells expressing the pluripotent markers TRA-1-60 and SSEA-4, flow cytometric analysis was performed, iPSC colonies were dissociated using TrypLE™ Express (Thermo Fisher Scientific) for 5 min at 37 °C. Dissociated cells were stained with the following antibodies: anti-human TRA-1-60-FITC (Millipore, FCMAB115) at 1:60 dilution and mouse anti-human SSEA-4-PerCPCy™5.5 (BD, 561565) at 1:20. Cell population was identified based on cell size and cell granularity. Single cells were discriminated using Forward Scatter area (FSC-A) and Forward Scatter Height (FSC-H) and live cells were gated from single cell population using DAPI nuclear staining (Partec). The cells were acquired using the BD LSRII flow cytometer (BD) and data analyzed using FlowJo software (Tree Star, Ashland, OR, USA). At least 10.000 events were collected for each analysis.

### In vivo test of pluripotency

For in vivo analysis of pluripotency via teratoma formation, 0.5 × 10^6^ iPSCs were resuspended in 50% Matrigel^TM^ (BD, 356234) and injected subcutaneously into both flanks of adult SCID male mice. Two animals were injected in each group. Following a period of 10 weeks, the mice were euthanized and the teratomae were excised, processed and sectioned according to standard procedures and stained for Weigert’s hematoxylin, Masson’s trichrome and Mayer’s hematoxylin and Eosin histological analysis. Sections (5–8 µm) were examined using bright field microscopy and stained tissue photographed as appropriate.

### Karyotyping and fingerprinting analysis

All cell lines were analyzed using Illumina CytoSNP analysis and the BlueFuse Multi 4.3 software (Illumina, San Diego, United States) according to standard protocols of the manufacturer.

### iPSC differentiation into hematopoietic progenitor cells

iPSCs maintained on Vitronectin™ in StemPro™ media were cut in homogeneous pieces using a STEMPRO® EZpassage™ tool (Thermo Fisher Scientific). Aggregates were resuspended in Stemline® II (Sigma-Aldrich) differentiation media supplemented with 1% penicillin/streptomycin and cultured in ultra-low attachment culture plates at 37 °C and 5% CO_2_ in a humidified incubator for 3 days to allow the formation of embryoid bodies (EBs). On day 3, EBs were dissociated using TrypLE™ Express for 10 min at 37 °C and transferred to tissue-culture treated wells to allow culture under monolayer conditions at 37 °C and 5% CO_2_ in a humidified incubator. Recombinant human BMP4 (day 0–2 10 ng/ml, day 2–16 20 ng/ml), VEGF (day 0–2 10 ng/ml, day 2–16 30 ng/ml), Wnt3A (10 ng/ml), GSK-3β Inhibitor VII (2 µM), Activin A (5 ng/ml), FGFα (10ng/ml), SCF (20 ng/ml), IGF-2 (10 ng/ml), TPO (10 ng/ml), β-estradiol (0.4 ng/ml), Heparin (5 µg/ml) and 3-isobutyl-1-methylxanthine (IBMX) (50 µM) were added to the differentiation media as previously described^18^. All cytokines and compounds were purchased from Peprotech except BMP4 and Wnt3A (R&D), GSK-3β Inhibitor VII (Calbiochem) and β-estradiol, Heparin and IBMX (Sigma-Aldrich).

### Detection of mesodermal and hematopoietic markers by flow cytometry

Differentiated cells were treated with 1X TrypLE™ Express for 5 min at 37 °C to obtain a single a cell suspension. Cell pellets were resuspended in FACS buffer (DPBS with FBS 2%) and cells were counted with a hemocytometer. A final amount of 1 × 10^5^ cells resuspended in 100 µl of FACS buffer with a dilution of 1:20 antibody was used for each analysis. The following cell surface antigens were analyzed for this study: KDR-PE (BD, 560494), CD34-APC (BD, 555824), CD43-FITC (Thermo Fisher Scientific, MHCD4301), CD41a-APCH7 (BD, 561422) and CD235a-BV421 (BD, 562938). Cells were washed using the BD FACS Lyse/Wash assistant (BD) and analyzed using BD LSRII flow cytometer (BD). Size and cell complexity were used to identify cell populations in a scatter graph representation. Single cells were discriminated using FSC-A and FSC-H and live cells were gated from single cell population using DAPI nuclear staining. Analysis of data was performed using FlowJo software (Tree Star Inc.). At least 10.000 events were collected for each analysis.

### Analysis of hematopoietic potential of hematopoietic progenitors by colony-forming unit assay

iPSC-derived hematopoietic progenitor cells were treated with 1X TrypLE™ Express for 5–10 min at 37 °C to obtain a single a cell suspension. TrypLE™ Express is diluted in PBS and cells were pelleted by centrifugation at 300 g for 3 min. Cell pellets were resuspended in FACS buffer (DPBS with 2% FBS) and cells were counted with a hemocytometer. A final amount of 6 × 10^4^ cells resuspended in 300 µl of FACS buffer and mixed with 3 ml of Methocult™ methylcellulose media enriched with recombinant cytokines (Stem Cell Technologies, 04435) and 1.5 ml were plated in duplicate in 35-mm dishes. Colonies were scored after 14 days of culture using light microscope according to standard criteria and averaged between the duplicate dishes.

### RNA isolation and reverse transcription PCR (RT-PCR)

RNA from iPSCs and fibroblasts at day-7 of SeV transduction used as positive Sendai control was extracted using the ReliaPrep^TM^ RNA Cell Miniprep System (Promega, Z6010) including DNase I treatment according to the manufacturer’s instructions. cDNA was generated from 1 µg of RNA using the GoScript^TM^ Reverse Transcription System (Promega, A5000) according to the manufacturer’s instructions.

For the PCR reaction mixture, 1 µl of cDNA produced from 1 µg of RNA was amplified using 10 µM dNTP mix, 5X Green GoTaq® Reaction Buffer and GoTaq® DNA Polymerase (5 u/µl) (Promega, M3175) and the following primers (10 µM) *SeV-OCT4* forward: 5′-CCCGAAAGAGAAAGCGAACCAG-3′;*SeV-OCT4* reverse: 5′-AATGTATCGAAGGTGCTCAA-3′; *SeV-SOX2* forward: 5′-ATGCACCGCTACGACGTGAGCGC-3′; *SeV-SOX2* reverse: 5′-AATGTATCGAAGGTGCTCAA-3′; *SeV-Klf4* forward: 5′-TTCCTGCATGCCAGAGGAGCCC-3′; *SeV-Klf4* reverse: 5′-AATGTATCGAAGGTGCTCAA-3′; *SeV-cMYC* forward: 5′-TAACTGACTAGCAGGCTTGTCG-3′; *SeV-cMYC* reverse: 5′-TCCACATACAGTCCTGGATGATGATG-3′; *SeV* forward: 5′-GGATCACTAGGTGATATCGAGC-3′; *SeV* reverse: 5′-ACCAGACAAGAGTTTAAGAGATATGTATC-3′. Oligonucleotides for the housekeeping gene- GAPDH were used as a positive control of the amplification reaction.

### DNA isolation and quantitative PCR for telomere length measurement

Genomic DNA was isolated using QiAamp DNA Mini Kit (Qiagen) according to manufacturer’s instructions. Telomere length was measured as abundance of telomeric template versus a single copy gene (36B4) as previously described^62^ using the following primers: TelA (5′-CGG TTT GTT TGG GTT TGG GTT TGG GTT TGG GTT TGG GTT-3′); TelB (5′-GGC TTG CCT TAC CCT TAC CCT TAC CCT TAC CCT TAC CCT-3′); 36B4F (5′-CAG CAA GTG GGA AGG TGT AAT CC 3′) and 36B4R (5′-CCC ATT CTA TCA TCA ACG GGT ACA A-3′). Three internal control DNA samples of known telomere length (10.4, 3.9 and 2 kb) were run within each plate to correct for plate–to-plate variation. Measurements were performed in triplicate. All PCRs were carried out on an Applied Biosystems 7900HT Fast Real Time PCR system with 384-well plate capacity. The intra-assay coefficient of variation was 2.7% while the inter-assay coefficient of variation was 5.1%.

### Telomere repeat amplification analysis for telomerase activity detection

Telomerase activity in iPSC and iPSC-derived hematopoietic progenitors was measured as previously described^63^. Final amount of 100 ng was used from protein lysate for each reaction. No protein/lysate samples were used as negative controls and 100, 10, 1, and 0.1 ng of protein lysate from Hela cells as positive controls.

### Analysis of DNA damage, proliferation and apoptosis by flow cytometry

Day 12 iPSC-derived hematopoietic progenitor cells were exposed to 2 M Hydroxyurea for 24 h and collected at different time points (0, 1, 3, 8, and 24 h). DNA damage, proliferation and apoptosis induction after HU-treatment was analyzed using a flow cytometric kit according to manufacturer’s instructions (BD). Briefly, at the specified time points after HU treatment, the cells were labeled with 50 µM BrdU and stained later with antibody anti-human CD43-FITC. The labeled cells were then fixed, permeabilised and labeled with anti-human γH2AX-Alexa Fluor®647, anti-human BrdU- PerCPCy™5.5 and anti-human Cleaved PARP (Asp214)-PE according to manufacturer’s instructions. DNA content for cell cycle analysis was determined by DAPI staining provided by the kit. Size and cell complexity were used to identify cell populations in a scatter graph representation and single cells were discriminated using FSC area (FSC-A) and FSC height (FSC-H). The cells were acquired using the BD LSRII flow cytometer (BD) and data analyzed using FlowJo software. At least 10.000 events were collected for each analysis.

### Exome sequencing analysis

DNA was extracted from 5 HLHS patient fibroblasts using QIAamp DNA Micro kit (Qiagen, Germantown, MD, 56304). Exome sequencing was performed by BGI. Exome capture was performed using Agilent SureSelect Human All Exon kit (V4). Libraries were constructed following the Illumina Paired-End Sequencing Library Preparation Protocol version 1.0.1 and then sequenced on the Illumina GAIIx platform with version 4 chemistry and version 4 flowcells. The sequencing reads were analyzed using the following workflow to identify variants in patient. The quality of sequencing reads was firstly checked with FastQC (Version 0.11.2)^64^ Low quality bases (Q < 20) on 3′ ends of reads were trimmed off using seqtk (Version 1.0)^65^. Duplicated reads were then removed with FastUniq (Version 1.1)^66^ before mapping to the human reference genome GRCh37 with BWA (Version 0.7.6.a)^67^. The alignments were refined with tools of the GATK suite (Version 3.2)^68^. Variants were called according to GATK Best Practice recommendations^69,70^, including recalibration. Freebayes (Version 1.0.1)^71^ was also used to call variants from the same set of samples. The variants called by Freebayes with total coverage ≥ 5, minor allele coverage ≥ 5 and variants call quality ≥ 20 were added to those identified by GATK. Non-synonymous exonic variants were subsequently filtered by quality and minor-allele frequency (MAF) reported in the 1000 Genomes project (2012 Feb release)^72^ and ESP6500^73^. Variants with MAF > 0.05 in either of the databases were excluded. ANNOVAR (Version 2014-07-22)^74^ was used for annotations and prediction of functional consequences. Deleterious in our study was defined by at least one of the predictors [SIFT or PolyPHEN2] and those that had alternative allele frequency less than 0.01 in both 1000 Genomes project and ESP6500 (Supplementary Table 2).

### Statistical analysis

Data are shown as mean ± S.E.M. from at least three independent experiments. The significance between means was determined with Multiple *t*-test using Holm-Sidak method and One-way ANOVA when Gaussian distribution was assumed and with Kruskal-Wallis test when Gaussian distribution was not assumed. Multiple comparisons test for comparison between particular pairs of control and patient groups. Statistically significant values were judged as follows: *P ≤ 0.05, **P ≤ 0.01, ***P ≤ 0.001, ****P ≤ 0.0001. Statistical analysis was performed using GraphPad Prism version 7.0 software and Minitab 17 statistical software.

## Electronic supplementary material

Supplemental Table 1

SupplementaL Table 2

Supplemental Figure 1

Supplemental Figure 2

Supplemental Figure 4

Supplemental Figure 5

Supplemental Figure 3

Supplemental Table 3

Legends supplemental information
