# Supplementary material for: iPSC modeling of severe aplastic anemia reveals impaired differentiation and telomere shortening in blood progenitors
Source: Cell Death Dis. 2018 Jan 26;9(2):128. doi: 10.1038/s41419-017-0141-1 (PMC5833558; doi:10.1038/s41419-017-0141-1)
Supplement: Supplementary file 1 — Supplemental Table 1 [file 41419_2017_141_MOESM1_ESM.docx]

| **Cell line** | **Cell type** | **Alteration (size Mb)** | **Locus** | **OMIM Disease-causing Genes** | **Karyotype** |
| --- | --- | --- | --- | --- | --- |
| **WT1** | Fibroblasts | - | - | - | 46XY |
|  | iPSC | - | - | - |  |
| **WT2** | Fibroblasts | - | - | - | 46XY |
|  | iPSC | - | - | - |  |
| **WT3** | Fibroblasts | - | - | - | 46XX |
|  | iPSC | - | - | - |  |
|  |  | - | - | - |  |
| **SAA1** | Fibroblasts | - | - | - | 46XY |
|  | iPSC | CN-LOH (1.3Mb) | 3q11.2 | - |  |
|  |  | CN-LOH (1Mb) | 7q22.1 | - |  |
| **SAA2** | Fibroblasts | CN-LOH (6.4Mb) | 11p11.12-q11 | - | 46XY |
|  | iPSC | CN-LOH (6.4Mb) | 11p11.12-q11 | - |  |
| **SAA3** | Fibroblasts | Deletion (1.5Mb) | 15q13.3 | *TRPM1, CHRNA7* | 46XX |
|  |  | Duplication (1.5Mb) | 16p13.11 | *NDE1, MYH11, ABCC6* |  |
|  | iPSC | Deletion (1.5Mb) | 15q13.3 | *TRPM1, CHRNA7* |  |
|  |  | Duplication (1.5Mb) | 16p13.11 | *NDE1, MYH11, ABCC6* |  |

**Supplemental Table 1**
