# Supplementary material for: iPSC modeling of severe aplastic anemia reveals impaired differentiation and telomere shortening in blood progenitors
Source: Cell Death Dis. 2018 Jan 26;9(2):128. doi: 10.1038/s41419-017-0141-1 (PMC5833558; doi:10.1038/s41419-017-0141-1)
Supplement: Supplementary file 2 — SupplementaL Table 2 [file 41419_2017_141_MOESM2_ESM.docx]

|  | **SAA1** | **SAA2** | **SAA3** |
| --- | --- | --- | --- |
| Exonic variants | 32202 | 33770 | 32977 |
| Rare exonic variants | 2740 | 3647 | 2820 |
| X-linked | 34 | 30 | 60 |
| Homozygous | 57 | 82 | 66 |
| Compound heterozygotes | 266 | 408 | 267 |
| Deleterious hits | 210 | 220 | 186 |

**Supplemental Table 2**
