# Supplementary figures and images for: iPSC modeling of severe aplastic anemia reveals impaired differentiation and telomere shortening in blood progenitors
Source: Cell Death Dis. 2018 Jan 26;9(2):128. doi: 10.1038/s41419-017-0141-1 (PMC5833558; doi:10.1038/s41419-017-0141-1)

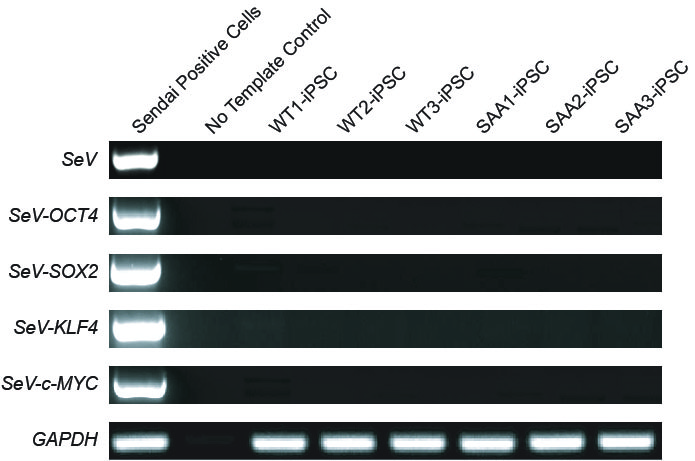

Supplement: Supplementary file 3 — Supplemental Figure 1 [file 41419_2017_141_MOESM3_ESM.jpg]

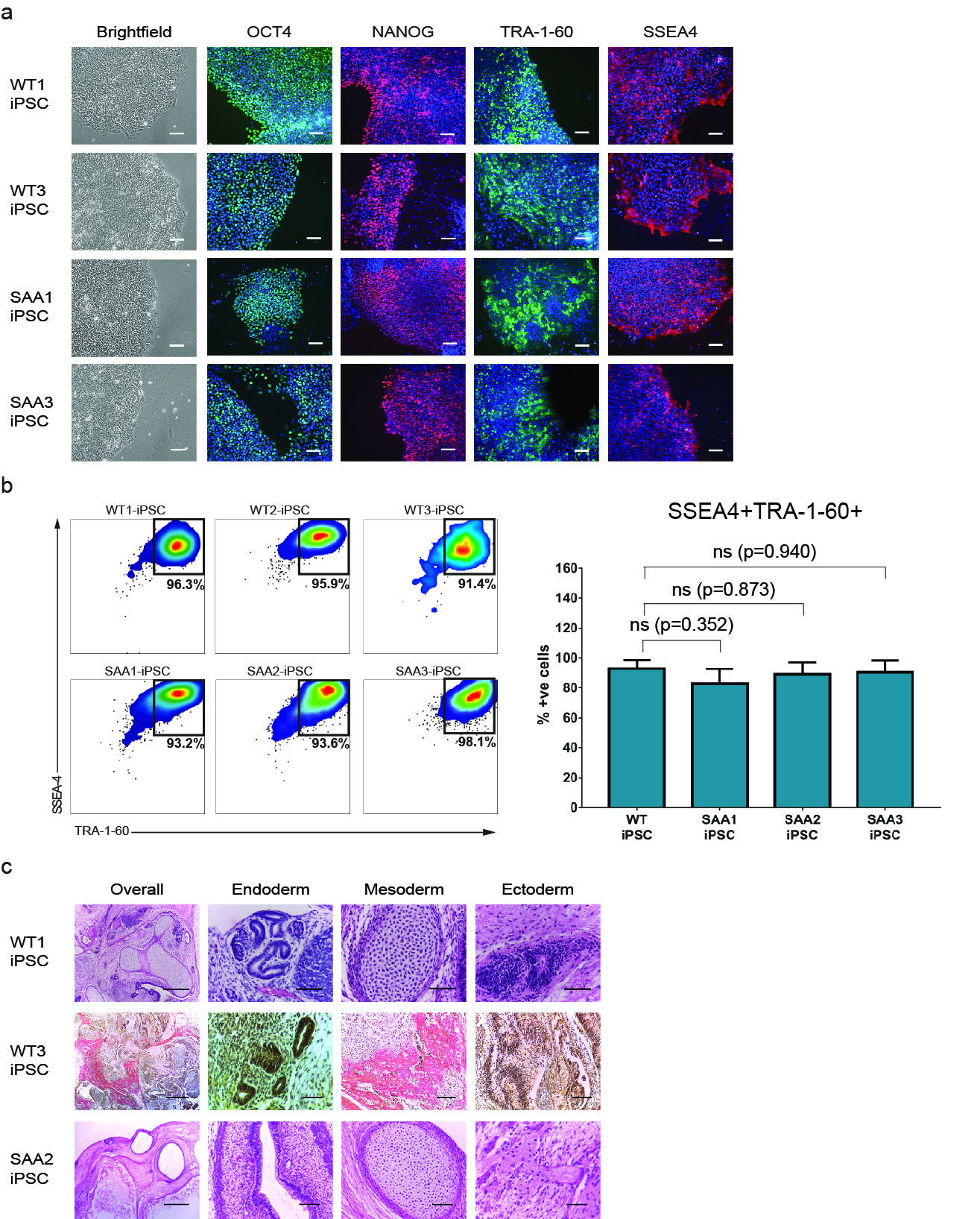

Supplement: Supplementary file 4 — Supplemental Figure 2 [file 41419_2017_141_MOESM4_ESM.jpg]

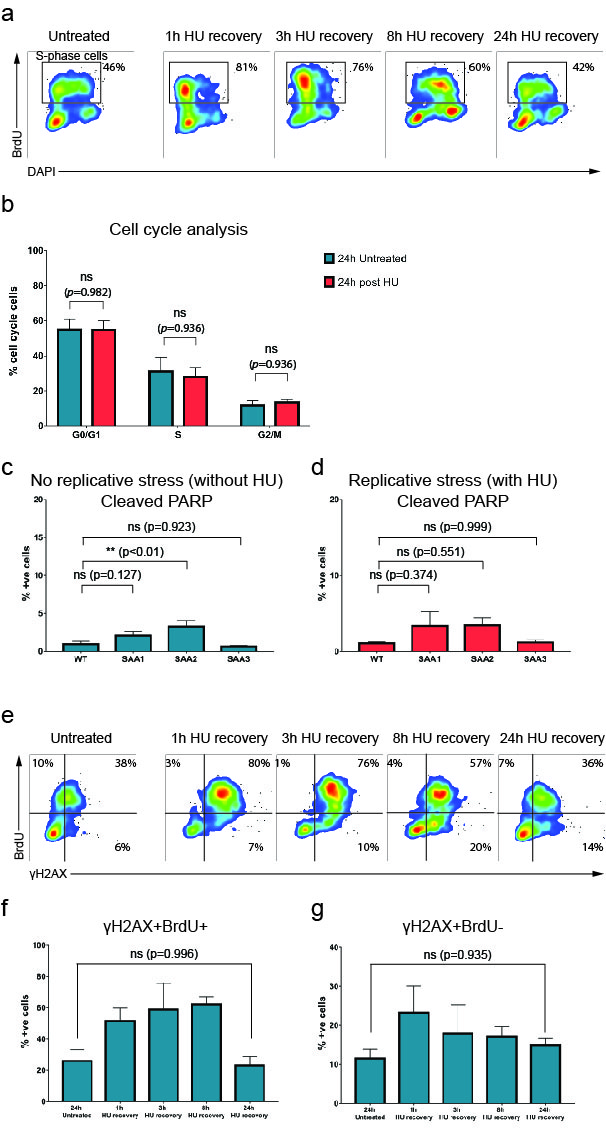

Supplement: Supplementary file 5 — Supplemental Figure 4 [file 41419_2017_141_MOESM5_ESM.jpg]

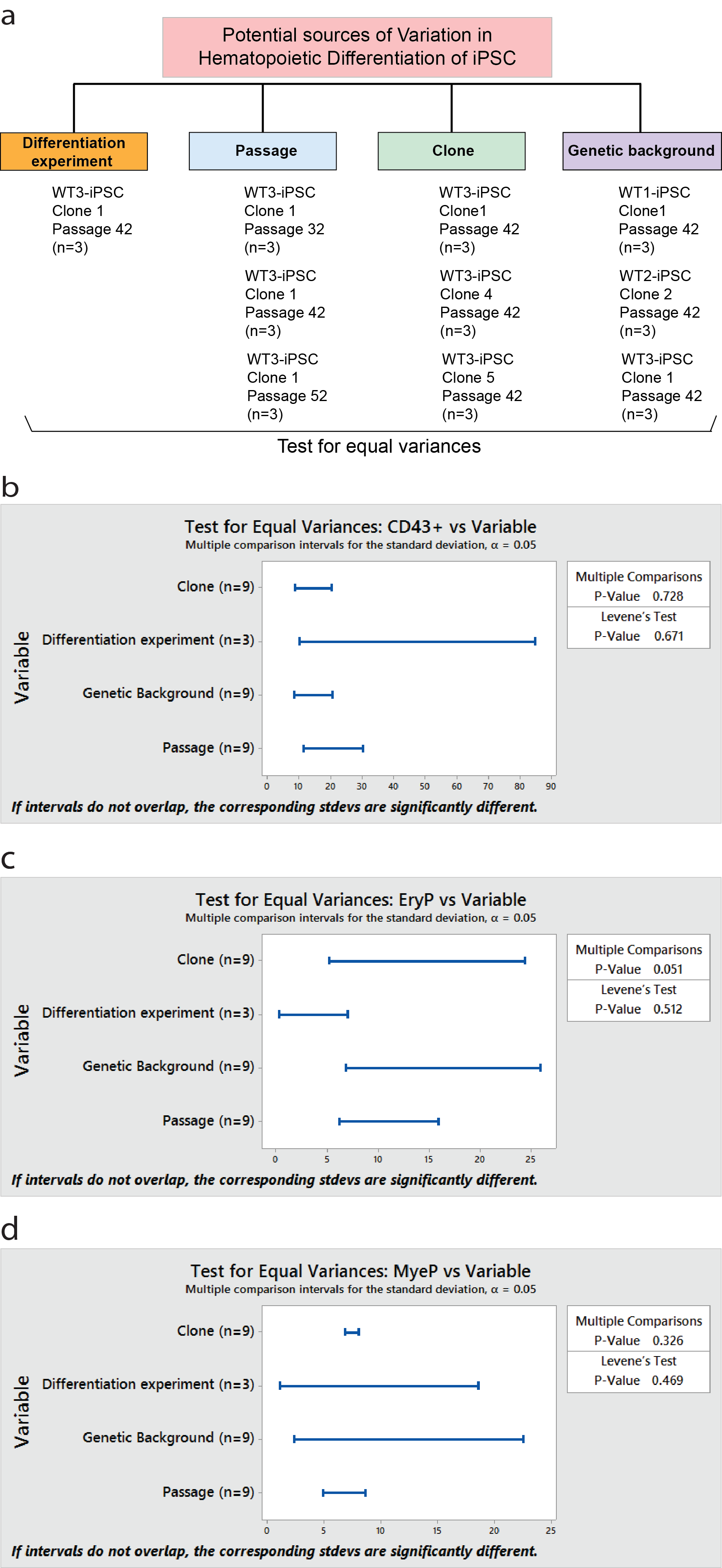

Supplement: Supplementary file 7 — Supplemental Figure 3 [file 41419_2017_141_MOESM7_ESM.png]
