# Supplementary material for: iPSC modeling of severe aplastic anemia reveals impaired differentiation and telomere shortening in blood progenitors
Source: Cell Death Dis. 2018 Jan 26;9(2):128. doi: 10.1038/s41419-017-0141-1 (PMC5833558; doi:10.1038/s41419-017-0141-1)
Supplement: Supplementary file 9 — Legends supplemental information [file 41419_2017_141_MOESM9_ESM.docx]

**Legends supplemental information**

**Supplemental Table 1.** Table providing detailed information on chromosomal alterations detected by SNP array in parental fibroblasts and iPSC in WT and SAA cell lines.

**Supplemental Table 2**. **Summary of exome sequencing performed on dermal skin fibroblasts obtained from the SAA patients**. A filtering strategy was used for each patient as follows:

1. Exonic variants were generated by removing Synonymous SNVs and non-exonic variants.
2. Rare exonic variants were generated by removing variants with alternative allele frequency in either 1000Genomes project / ESP6500 / cg69 / inHouseExomes / InHouse GATK Exomes greater than 0.05
3. Rare interested hits were generated if they had both 1000Genomes Freq <= 0.01 and ESP6500 Freq <= 0.01.
4. X linked variants were generated by removing variants that were not on chromosome X.
5. Homozygous variants were generated by removing variants that were not on autosomal chromosomes, compound heterozygotes and heterozygous variants.
6. Compound heterozygotes were generated by keeping genes with two or more rare variants.
7. Deleterious variants were generated by selecting variants that were predicted Deleterious by at least one of the predictors (SIFT or PolypHEN2) and those that had alternative allele frequency less than 0.01 in both 1000Genomes project and ESP6500.

**Supplemental Table 3**. List of deleterious variants identified in SAA patients by exome sequencing analysis.

**Supplemental Figure 1.** Expression of reprogramming transgenes was analyzed by RT-PCR using transgene-specific primers in WT and SAA-iPSC. Fibroblasts at day 7 post transduction were used as a positive control.

**Supplemental Figure 2.** (**a**) Brightfield images of control and SAA-iPSC colonies displaying typical ESC-like morphology and immunostaining of WT and SAA-iPSC colonies with pluripotency markers. DAPI staining is shown in blue. Scale bars, 100µm. (**b**) Representative images of flow cytometric analysis (left hand panel) and schematic graphs (right hand panel) of SSEA-4 and TRA-1-60 expression in WT and SAA cell lines on day 0. One-way ANOVA with Dunnett’s multiple comparison test was used for statistical comparison between WT and SAA cell lines. Data is presented as mean of at least 3 independent experiments +/- S.E.M. . Data for all control cell lines is averaged in one group (WT); (**c**) Histological analysis of representative teratomae generated from the WT and SAA-iPSC lines displaying trilineage differentiation. Scale bars, overall 500µm, ectoderm 100µm, mesoderm 200µm, ectoderm 100µm.

**Supplemental Figure 3.** (**a**) Schematic representation of the experimental design to analyze variation among the different variables considered in the iPSC hematopoietic differentiation (experiment, passage, clone and genetic background) using the test for equal variances with multiple comparisons method for the different populations of progenitors: (**b**) Hematopoietic progenitor population (CD43+); (**c**) Erythroid progenitor (EryP) population; (**d**) Myeloid progenitor (MyeP) population.

**Supplemental Figure 4**. Reduced hematopoietic colony forming ability of SAA-iPSC-derived hematopoietic progenitors at day 16 of differentiation.

**Supplemental Figure 5.** (**a**) Flow cytometric analysis of BrdU and DAPI incorporation in untreated, 1 hour HU recovery, 3 hour HU recovery, 8 hour HU recovery and 24 hour HU recovery WT-iPSC-derived hematopoietic progenitor cells; (**b**) Analysis of cell cyle in untreated (dark blue bars) and 24 hour HU recovery (red bars) WT-iPSC-derived hematopoietic progenitor cells. Multiple t-test using Holm-Sidak method was used for statistical comparison between untreated and 24 hour HU recovery WT cells; (**c**) Analysis of cleaved PARP in WT and SAA-iPSC-derived hematopoietic progenitor cells in non-replicative stress conditions. One-way ANOVA with Dunnett’s multiple comparison test was used for statistical comparison between WT and SAA cell lines; (**d**) Analysis of cleaved PARP in WT and SAA-iPSC-derived hematopoietic progenitor cells in replicative stress conditions. One-way ANOVA with Dunnett’s multiple comparison test was used for statistical comparison between WT and SAA cell lines; (**e**) Flow cytometric analysis of BrdU incorporation and H2AX detection in untreated, 1 hour HU recovery, 3 hour HU recovery, 8 hour HU recovery and 24 hour HU recovery WT-iPSC-derived hematopoietic progenitor cells; (**f**) Analysis of γH2AX in BrdU+ untreated, 1 hour HU recovery, 3 hour HU recovery, 8 hour HU recovery and 24 hour HU recovery WT-iPSC-derived hematopoietic progenitor cells; (**g**) Analysis of γH2AX in BrdU- untreated, 1 hour HU recovery, 3 hour HU recovery, 8 hour HU recovery and 24 hour HU recovery WT-iPSC-derived hematopoietic progenitor cells. **b-d, f-g**: data is presented as mean of at least 3 independent experiments +/- S.E.M. Data for all control cell lines is averaged in one group (WT).
